# Genome wide effects of oleic acid on cultured bovine granulosa cells: evidence for the activation of pathways favoring folliculo-luteal transition

**DOI:** 10.1186/s12864-021-07817-6

**Published:** 2021-06-29

**Authors:** Vengala Rao Yenuganti, Dirk Koczan, Jens Vanselow

**Affiliations:** 1 Animal Biology Department, School of Life Sciences, University of Hyderabad, Hyderabad, Telagana India; 2 Institute for Immunology, University of Rostock, 18055 Rostock, Germany; 3 Institute of Reproductive Biology, Leibniz Institute for Farm Animal Biology (FBN), Wilhelm-Stahl-Allee 2, 18196 Dummerstorf, Germany

**Keywords:** Gene expression, Free fatty acids, Estradiol, Angiogenesis, Metabolic stress, Cell culture

## Abstract

**Background:**

Metabolic stress, as negative energy balance on one hand or obesity on the other hand can lead to increased levels of free fatty acids in the plasma and follicular fluid of animals and humans. In an earlier study, we showed that increased oleic acid (OA) concentrations affected the function of cultured bovine granulosa cells (GCs). Here, we focus on genome wide effects of increased OA concentrations.

**Results:**

Our data showed that 413 genes were affected, of which 197 were down- and 216 up-regulated. Specifically, the expression of FSH-regulated functional key genes, *CCND2*, *LHCGR*, *INHA* and *CYP19A1* and 17-β-estradiol (E2) production were reduced by OA treatment, whereas the expression of the fatty acid transporter *CD36* was increased and the morphology of the cells was changed due to lipid droplet accumulation. Bioinformatic analysis revealed that associated pathways of the putative upstream regulators “FSH” and “Cg (choriogonadotropin)” were inhibited and activated, respectively. Down-regulated genes are over-represented in GO terms “reproductive structure/system development”, “ovulation cycle process”, and “(positive) regulation of gonadotropin secretion”, whereas up-regulated genes are involved in “circulatory system development”, “vasculature development”, “angiogenesis” or “extracellular matrix/structure organization”.

**Conclusions:**

From these data we conclude that besides inhibiting GC functionality, increased OA levels seemingly promote angiogenesis and tissue remodelling, thus suggestively initiating a premature fulliculo-luteal transition. In vivo this may lead to impeded folliculogenesis and ovulation, and cause sub-fertility.

**Supplementary Information:**

The online version contains supplementary material available at 10.1186/s12864-021-07817-6.

## Background

Dairy cows frequently suffer from negative energy balance (NEB) after parturition. Under these metabolic conditions, the serum levels of non-esterified fatty acids (NEFAs) become elevated and can negatively affect the reproductive performance [1–3]. Also in obese women, it has been reported that abnormal NEFA levels and lipid dysregulation are associated with fertility problems [4–6]. During NEB in lactating dairy cows, fat from adipose tissue is mobilized to meet the animal’s energy requirements. Consequently, levels of free fatty acids (FFAs) like palmitic acid (PA, 16:0), stearic acid (SA, 18:0), oleic acid (OA, 18:1) and of β-hydroxybutyric acid, increase in the plasma and follicular fluid [3, 7] and can negatively affect milk production and cause increased vulnerability to infections, various metabolic diseases and reduced fertility [8–10]. Also short-term fasting increases the levels of different fatty acids especially of OA in the follicular fluid [11]. Various studies showed that high levels of FFAs are indicators of abnormal lipid metabolism and can affect growth, differentiation and metabolism of cells by altering the gene expression levels [12–14]. Also in granulosa cells (GCs) it has been found that free fatty acids like PA, SA and OA affect cell survival in both humans and bovine [15, 16]. In our previous studies, we showed in a bovine GC culture model that OA affects the cell morphology and reduces expression of genes that are involved in 17-β-estradiol (E2) production and gonadotropin signalling as *CYP19A1*, FSHR and LHCGR [17, 18]. Also the GC identity marker FOXL2 was down-, whereas the marker of sertoli cells SOX9 was up-regulated [19]. Intriguingly, we could show in addition that OA elicited partly opposing effects compared to PA and SA if applied individually [18]. During the present genome-wide approach we selected to study OA effects because it was found at highest concentration in animals after fasting as compared to PA and SA [11] and it was shown to elicit strong and reproducible effects on cultured E2 producing GCs [17, 18].

To elucidate the pathways and upstream molecules that are involved in GC dysfunction under OA treatment we studied effects of OA on global gene expression and analyzed effects on signaling pathways, upstream regulators and biological processes.

## Results

### Oleic acid induced differentially expressed genes

**Fig. 1:**
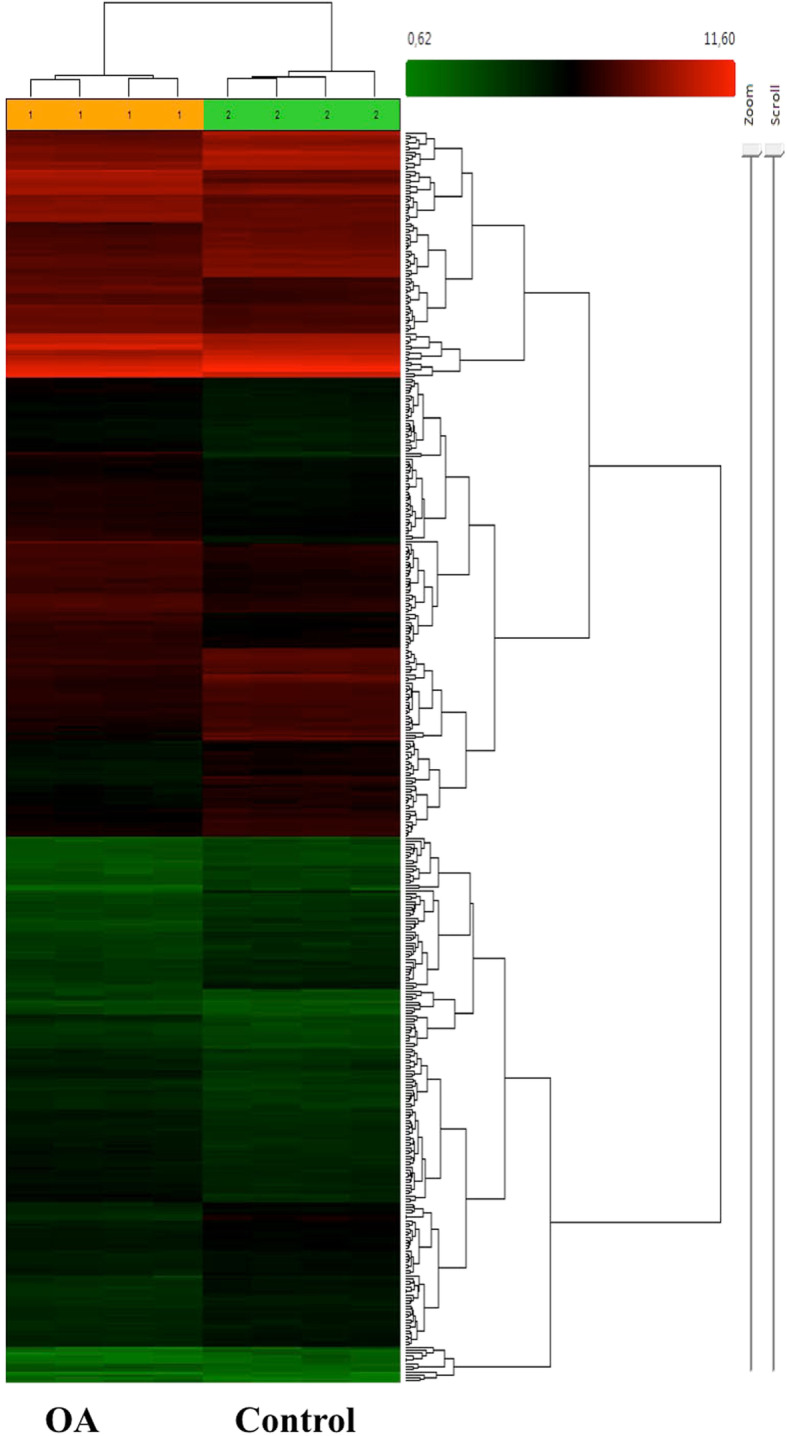
Microarray analysis. The figure shows a heat map analysis of DEGs. OA treated and untreated GCs were analyzed after 8 days in culture under stimulatory conditions with FSH and IGF-1 supplementation throughout the time of culture. The cells were exposed to OA or vehicle control from day 2. Data were obtained from four samples in each group

Microarray analysis of OA vs. vehicle treated GCs after 8 days in culture resulted in 413 differentially expressed genes (DEGs, Supplementary Table 1). According to the statistical criteria used (FC > |1.5|, ANOVA *p* < 0.05, and FDR q < 0.05) OA treated and vehicle treated control samples (*n* = 4 in each group) were clearly separated by hierarchical cluster analysis (Fig. 1). Out of the 413 DEGs, 197 were down- and 216 genes up-regulated. *ALDH1A1* (FC-6.15) was the most up-regulated gene, whereas *CYP19A1* (FC − 4.68) was strongly down-regulated by OA (Table 1).

**Table 1:** Top 24 DEGs affected by OA treatment of cultured GCs

#	Gene Symbol	Fold change	***p***- value
1	*ALDH1A1*	6.15	0.000001
2	*ANGPTL4*	5.69	2.10E-09
3	*PAG11*	4.4	7.01E-08
4	*CD36*	3.65	3.33E-08
5	*SLC38A4*	3.59	0.000044
6	*PLA2R1*	3.39	0.000014
7	*KIAA1199*	3.3	1.98E-08
8	*DHRS9*	3.08	8.76E-07
9	*LOC537017*	2.77	0.000018
10	*ANKRD1*	2.73	1.77E-09
11	*CD44*	2.73	5.76E-07
12	*EFEMP1*	2.69	3.33E-07
13	*CYP19A1*	-4.68	0.000004
14	*CNIH3*	−4.03	4.60E-08
15	*JAK3*	−3.38	2.94E-08
16	*TLL2*	−3.31	2.66E-07
17	*CDH20*	−3.07	0.000004
18	*SUSD4*	−2.91	0.000003
19	*NOS2*	−2.9	2.63E-07
20	*AFMID*	−2.87	6.78E-07
21	*QRFPR*	−2.77	2.00E-06
22	*SLC28A2*	−2.76	2.40E-05
23	*CMBL*	−2.71	2.00E-06
24	*KCND2*	−2.68	1.40E-05

*DEGs* Differentially expressed genes, *GCs* Granulosa cells, *OA* Oleic acid

### Real -time RT-PCR validation of differentially expressed genes

**Fig. 2:**
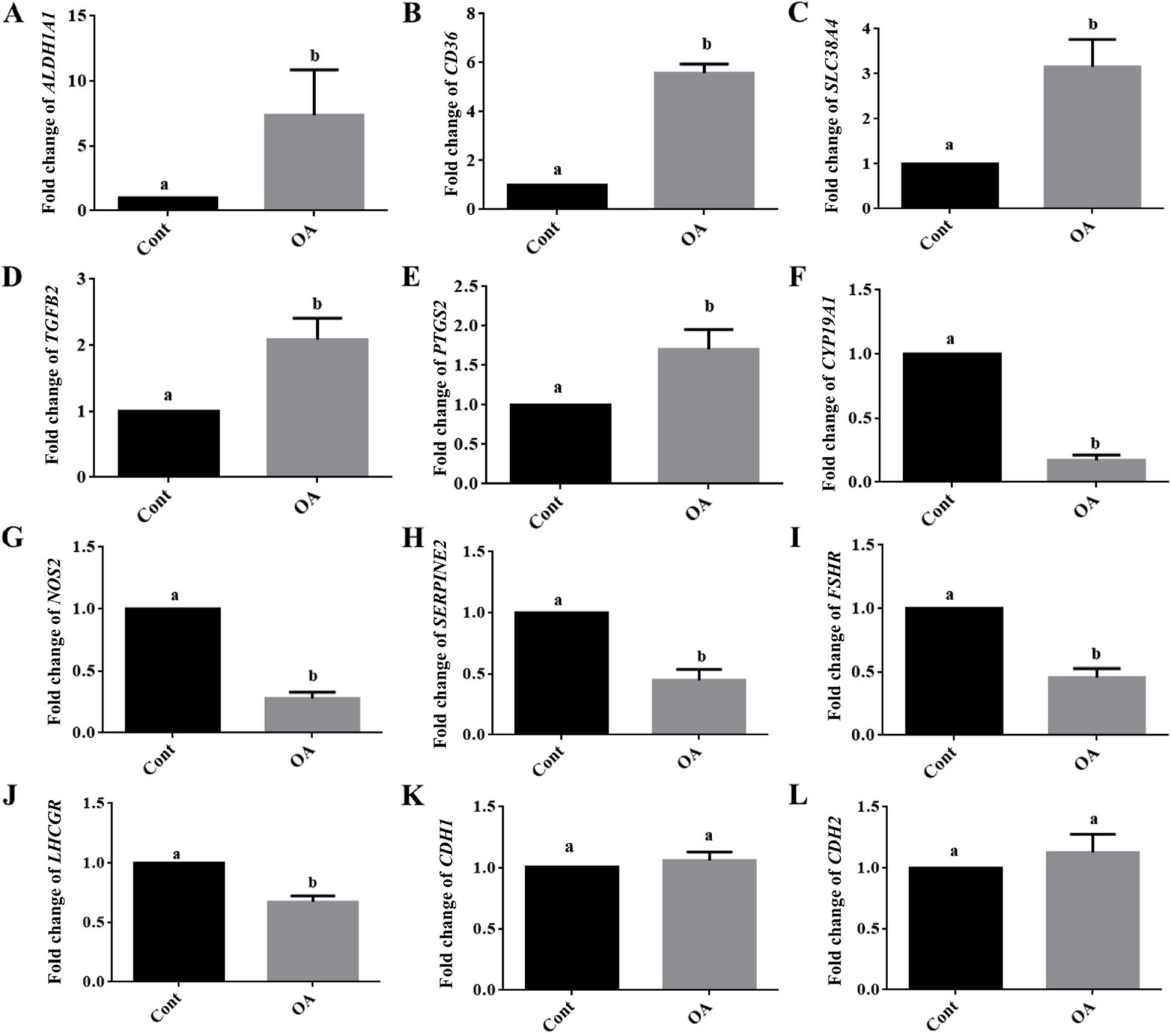
Microarray data validation by real-time RT PCR. The abundance of different transcripts from OA affected and non-affected genes were analyzed by real-time RT-PCR. The results clearly showed that *ALDH1A1, CD36, SLC38A4, TGFB2* and *PTSG2* were up-regulated (A-E), *CYP19A1*, *NOS2, SERPINE2, FSHR*, *LHCGR*, (F-J) were down-regulated and *CDH1* and *CDH2 (K, L)* were unaffected by OA treatment thus confirming the microarray data. GC samples were analyzed after 8 days in culture under stimulatory conditions with FSH and IGF-1 supplementation throughout the time of culture and OA or vehicle treatment from day 2. Different letters indicate significant differences relative to the respective controls, which were set to one (mean fold change ± standard deviation, *P* < 0.05, one-way ANOVA from four independent experiments). Cont denotes vehicle controls and OA denotes oleic acid treatment

**Fig. 3:**
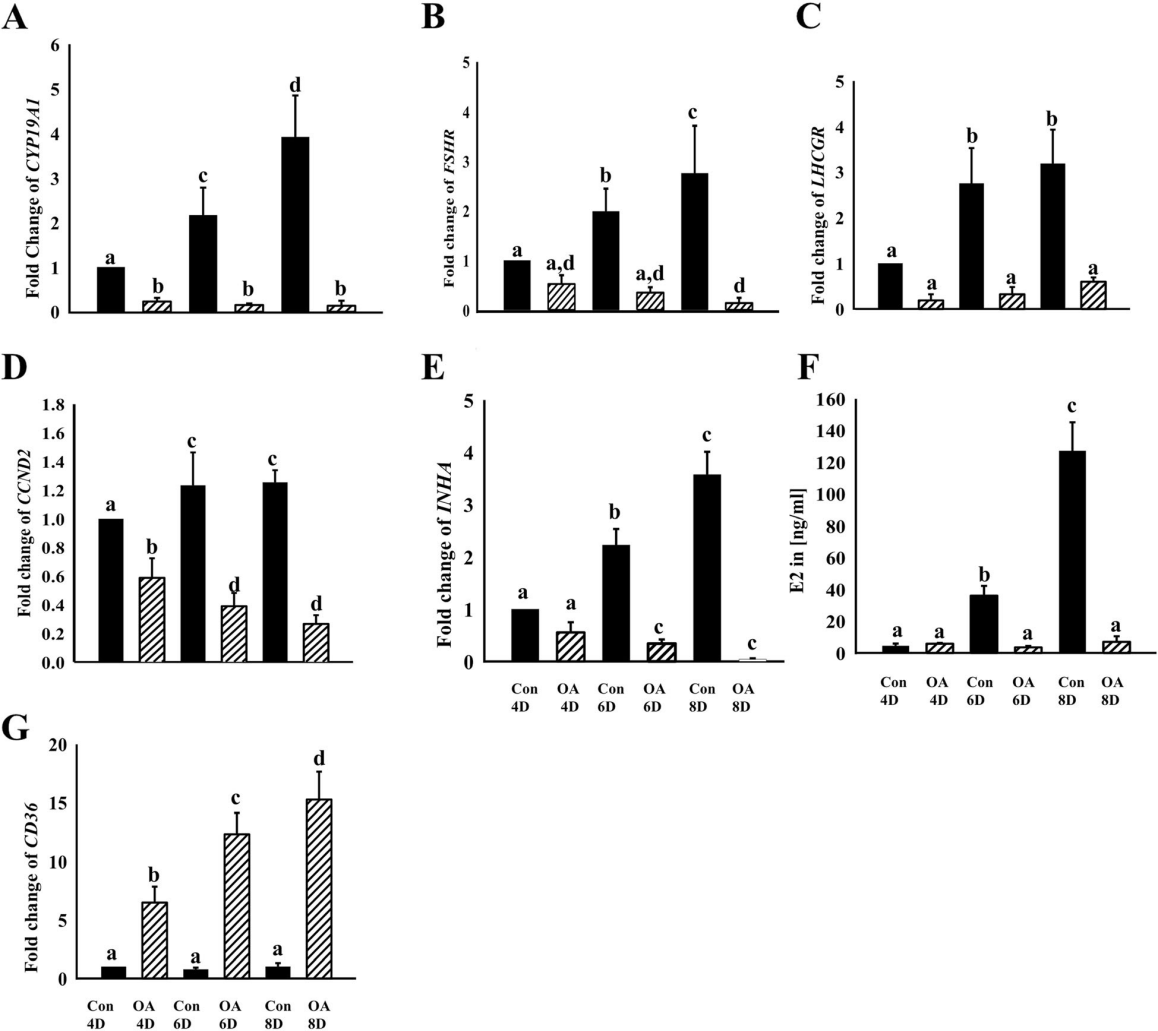
Time dependent effects of OA on selected transcripts and E2 production. GCs were cultured under stimulatory conditions with FSH and IGF-1 supplementation throughout the time of culture. After 2 days, the cells were treated in addition with OA or vehicle control. Media with all respective supplements were replaced every other day. Transcript abundance of different genes (**A**, **B**, **C**, **D**, **E**, **G**) and levels of estradiol (**F**) were determined after 4, 6 and 8 days in culture in OA or vehicle treated GCs. OA treated samples showed reduced transcript abundance of *CYP19A1, FSHR, LHCGR, CCND2, INHA FSHR,* but increased levels of *CD36*. Different letters indicate significant differences of fold changes (mean fold change ± standard deviation, *P* < 0.05, one-way ANOVA from three independent experiments) relative to untreated, but FSH and IGF-1 stimulated cultures after day 4, which were set to one. Con denotes vehicle controls and OA denotes oleic acid treatment

From the DEGs we selected functionally interesting up-regulated (*ALDH1A1*, *CD36*, *SLC38A4*, *TGFB2* and *PTGS2*), down-regulated (*CYP19A1*, *NOS2*, *SERPINE2*, *FSHR* and *LHCGR*) and non-regulated genes (*CDH1* and *CDH2*) for validation by real-time RT-PCR. The results clearly indicated similar OA effects after 8 days in culture thus confirming the microarray data (Fig. 2). An additional analysis also including earlier time points (4, 6 and 8 days in culture) indicated that the transcript abundance of several genes (*CYP19A1, FSHR, LHCGR*) remarkably increased under control conditions, but not in the presence of OA (Fig. 3). Instead, the abundance of some transcripts was even reduced by OA (*CCND2, INHA*). The E2 accumulated over time, but was nearly absent in OA treated samples. In contrast, *CD36* was only expressed at very low levels under control conditions, but was largely stimulated by OA treatment.

### Bioinformatics analysis of differentially expressed genes

IPA analysis of DEGs predicted that OA might modulate different Upstream Regulators, Canonical Signaling Pathways, and Functions and Diseases. 98 “Upstream Regulators” were affected with z-scores >|2|, out of which 74 were activated and 24 inhibited (Supplementary Table 2). OA activated the Upstream Regulators “TGFB1”, “Cg (choriogonadotropin)”, “Lipopolysaccharide”, “Rosiglitazone”, “Tgf beta”, “NFkB (complex)” and “HIF1A” etc., and inhibited “SB203580”, “FSH”, “PD98059”, “SMAD7”, “Y 27632” and “NR5A1” etc.

IPA analysis also predicted many affected Canonical Pathways. Top scores were indicated for “Hepatic Fibrosis / Hepatic Stellate Cell Activation”, “Ovarian Cancer Signaling”, “LPS/IL-1 Mediated Inhibition of RXR Function”, “Atherosclerosis Signaling” and “LXR/RXR Activation” (Supplementary Table 3).

Moreover IPA analysis of differentially expressed genes predicted 69 possibly affected diseases and functions. The most significant were “Cellular Movement”, “Cancer”, “Organismal Injury and Abnormalities”, “Cardiovascular System Development and Function” and “Organismal Development” (Supplementary Table 4).

Gene Ontology (GO) term analysis using the WebGestalt tool, showed that among others, down-regulated genes were associated with the terms “reproductive structure/system development”, “ovulation cycle process” and “regulation of follicle-stimulating hormone secretion” (Table 2 and Supplementary Figure 1). Simultaneously, the analysis of GO terms associated with up-regulated genes revealed effects on “circulatory system development”, “vasculature development”, “cardiovascular system development”, “extracellular matrix/structure organization/organization”, “angiogenesis”, “blood vessel morphogenesis” and “cell adhesion” (Table 2 and Supplementary Figure 2).

**Table 2:** Affected Gene Ontology (GO) terms according to WebGestalt Over-Representation Analysis

Regulation	GO	Name	gNum	FDR
Down	0048608	Reproductive structure development	16	1.21E-02
Down	0061458	Reproductive system development	16	1.21E-02
Down	0048468	Cell development	39	1.21E-02
Down	0003006	Developmental process involved in reproduction	19	2.32E-02
Down	0022602	Ovulation cycle process	7	2.32E-02
Down	0046880	Regulation of follicle-stimulating hormone secretion	3	2.32E-02
Down	0046881	Positive regulation of follicle-stimulating hormone	3	2.32E-02
Down	0007167	Enzyme linked receptor protein signaling pathway	24	2.82E-02
Down	0032278	Positive regulation of gonadotropin secretion	3	2.82E-02
Down	0046884	Follicle-stumulating hormone secretion	3	2.82E-02
Up	0072359	Circulatory system development	42	4.61E-10
Up	0048646	Anatomical structure formation involved in morphogenesis	39	6.32E-08
Up	0001944	Vasculature development	30	9.44E-08
Up	0072358	Cardiovascular system development	30	9.82E-08
Up	0043062	Extracellular structure organization	22	1.24E-07
Up	0030198	Extracellular matrix organization	22	1.24E-07
Up	0009888	Tissue development	53	1.45E-07
Up	0001525	Angiogenesis	24	1.82E-07
Up	0048514	Blood vessel morphogenesis	26	2.48E-07
Up	0007155	Cell adhesion	50	7.64E-07

### Time dependent effects of OA on GC morphology

**Fig. 4:**
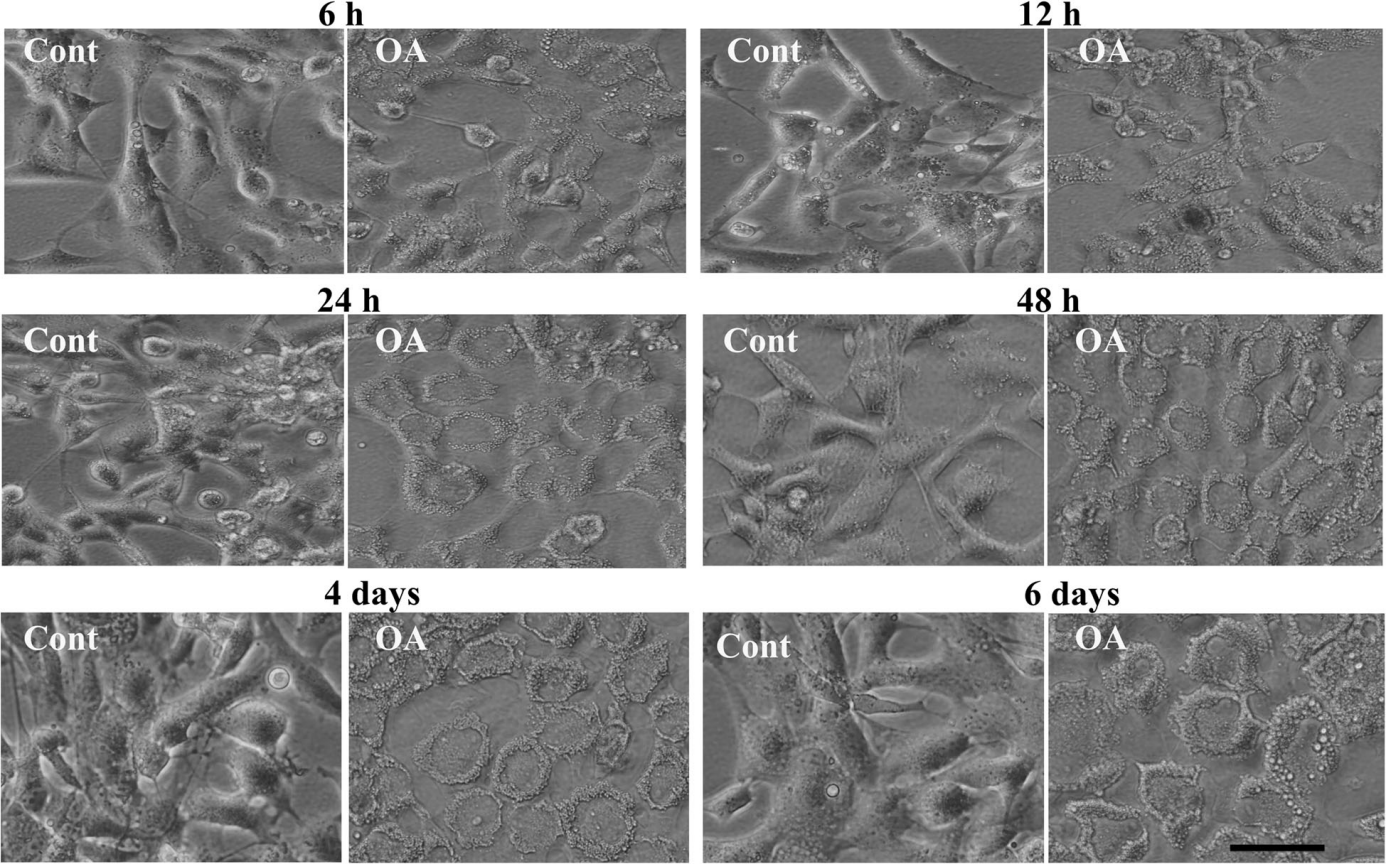
Time dependent morphological changes of GCs after OA treatment. Following 2 days of stimulatory culture conditions with FSH and IGF-1 supplementation GCs were treated with OA or vehicle control in addition and photographed 6 h, 12 h, 24 h, 48 h, 4d and 6 later to study the OA induced changes of morphological features. A continuous change of the morphology from a fibroblast-like towards a flat, foam cell-like morphology can be seen. Cont denotes vehicle controls and OA denotes oleic acid treated cells. Scale bar = 50 μm

After treatment with OA, cultured GCs were evaluated under a microscope 6 h, 12 h, 24 h, 48 h, 4 days and 6 days after treatment. The cell morphology showed that OA induced morphological changed from a more fibroblast-like to a foam-cell-like structure in a time dependent manner (Fig. 4). In a previous study, we could show that this morphological change is mainly due to considerable lipid droplet accumulation [17].

## Discussion

Bioinformatic analysis of DEGs by IPA suggests that various upstream regulators were affected by OA treatment in cultured bovine GCs. Most interesting, FSH and NR5A1 (also known as steroidogenic factor 1, SF-1) were among the inhibited regulators. Both factors and associated pathways are known to play significant roles during folliculogenesis [20–23]. Down-regulation of FSH signaling is in line with our previously published data indicating that in particular the abundance of FSHR transcripts and thus the corresponding pathways and genes are down regulated by OA treatment [17]. This may result in the observed prevention of up-regulation of the functional key genes *CYP19A1*, *CCND2*, *LHCGR* and *INHA* and of E2 production from day 4 to the end of the experiment (see Fig. 3). In addition to this, also Over-Representation Analysis of down-regulated genes revealed that most of these are involved in female reproduction related processes such as “reproductive structure/system development”, “ovulation cycle process”, “(positive) regulation of follicle-stimulating hormone/gonadotropin (secretion)” (see Table 2). Thus, these data indicate that the presence of OA compromised GC functionality and suggest that OA may impair folliculogenesis and the ovarian cycle in cows. This is also supported by the observation of morphological changes in OA treated GCs showing lipid droplet accumulation. Also in human lipid droplet accumulation in GCs have been correlated with fertility problems [24]. Also recent in vivo data suggest that increased NEFA levels and in particular increased OA concentrations in the follicular fluid have detrimental effects on follicle growth and even prevent ovulation [18, 25]. However, the role of OA seems complex and is still controversial. Some studies also clearly demonstrated positive effects on oocytes and during early embryo development as well as protective actions against lipotoxicity [26, 27].

Among the activated upstream regulators the most interesting were TGFB1, Cg (choriogonadotropin), Tgf beta, NFkB, HIF1A, EDN1 and WNT3A (see Supplementary Table 2). The significant activation of the Cg pathway suggests that LH signaling was induced by increased OA concentrations via the luteinizing hormone/choriogonadotropin receptor (LHCGR) pathway. Together with the down-regulated GC functionality, this suggests that the cells are driven towards luteinization. In vivo the preovulatory LH surge induces a rapid and profound molecular transformation of follicular cells termed luteinization in particular in GCs [28–30]. These changes (down-regulation of E2 production and of follicular marker transcripts) seem to be at least partly mimicked by OA in cultured GCs. However, this transformation is certainly not complete. Luteinization of GCs is not only associated with down-regulation of E2 but in addition with up-regulation of progesterone (P4) production. This has been clearly shown in vivo after the preovulatory LH surge [28, 31]. However, we could show in our previous studies that OA treatment of cultured GCs is clearly associated with decreased E2 but not with increased P4 production. Contrary, P4 production was even down-regulated [17, 18].

Interestingly, also TGFB1, Tgf beta, NFkB, HIF1A and WNT3A were indicated as activated by OA treatment. According to previous studies these factors and associated pathways are involved in the regulation of the Epithelial-to-Mesenchymal Transition (EMT) in several cell types including ovarian cells [32]. Activation of these regulators thus suggests EMT up-regulation by OA treatment. Partial EMT is a characteristic feature of GCs entering the folliculo-luteal transformation [33]. It is also quite noticeable that EDN1 is among the activated upstream regulators. On one hand, endothelins are known to be involved in angiogenic processes [34–36] and on the other hand, the formation of new vasculature is an essential feature of the developing corpus luteum [37, 38]. EDN1, EDN2 and EDN3 as well as endothelin receptors type A and B (EDNRA, ENDRB) are present in bovine GCs [39] as well as in our cultured GCs whereby EDNRA is significantly down- and ENDRB significantly up-regulated (see Supplementary Table 2). Taken together, down-regulation of GC functionality and simultaneous activation of the endothelin pathway and of GO terms related to angiogenesis, vascularization and morphogenic processes (e.g. Anatomical structure formation, Extracellular structure/matrix organization, see Table 2) support our hypothesis that OA treatment induces a luteal-like transformation in cultured GCs. Not least, also the observed lipid droplet accumulation in OA treated cells suggests their more luteal-like character since lipid droplets are a major feature of steroidogenic luteal cells [40].

Intriguingly, IPA analysis of affected “Canonical Pathways” and “Cell functions and Diseases” provided also evidence that cancer-related signaling might be promoted by OA treatment (see Supplementary Tables 3 and 4). In addition, the up-regulation of genes involved in angiogenic and morphogenic processes can be interpreted as supportive of the alternative hypothesis that increased OA concentrations facilitate tumor initiation and progression. However, cancer studies are certainly overrepresented in databases used by IPA and other bioinformatic tools. This may lead to a biased output favoring pathway and regulators involved in cancerogenesis. In addition, the observed prevalence of GC cancer in cattle is very low (0.5–0.75%) according to several studies [41, 42]. Accordingly, this interpretation of our data must be treated with caution and certainly needs additional experimental evidence.

## Conclusions

Based on our results and on studies from previous reports we propose that OA induces fatty acid uptake and lipid droplet accumulation in GCs and may inhibit FSH signaling and affect the expression of many genes, which are involved in GC functionality. On the other hand, genes and pathways involved in angiogenic and morphogenic processes are up-regulated thus suggesting that OA may activate pathways favoring folliculo-luteal transition.

## Methods

### Culture of granulosa cells

Isolation and culture of GCs were done as previously described [17, 43, 44]. Briefly, bovine ovaries were collected from a commercial slaughterhouse (Danish Crown Teterower Fleisch GmbH, Teterow). GCs were aspirated from small to medium follicles (2-6 mm diameter) that contain clear antral fluid with an 18-gauge needle in PBS with antibiotics. The percentage of viable cells was determined in a hemocytometer by using the trypan blue exclusion method. Routinely, 1.25 × 10^5^ viable cells were seeded on 24-well collagen R (Serva, Heidelberg, Germany) coated plates per 0.5 ml of basal α-MEM (Merck/Biochrom, Berlin, Germany) without serum but supplemented with 25 ng/ml IGF-1, 20 ng/ml FSH and 2 μM androstenedione (Sigma Aldrich, Steinheim, Germany) to induce E2 production. Conditioned media were replaced with fresh media including all respective supplements every other day. Two days after seeding 400 μM of OA (Sigma Aldrich, Steinheim, Germany) dissolved in ethanol as described earlier [17] was added with the first change of media (i.e. after 2 days in culture). The concentration of 400 μM was selected, because similar levels have been found in vivo under NEB conditions and this concentration has been also shown to efficiently affect gene expression and hormone production in GCs [11, 17]. To allow the cells to recover from plating stress and to re-acquire GC-like morphological and physiological features (expression of marker genes and E2 production) the cells were cultured for 8 days [44] for microarray analysis. In addition, early alterations of the abundance of selected marker transcripts and of E2 concentrations in spent media were also studied after 4, 6 and 8 days. Early morphological changes were analyzed 6 h, 12 h, 24 h, 48 h, 4th day and 6th day after addition of OA by using a Nikon TMS-F inverted microscope.

### RNA isolation and cDNA synthesis

RNA isolation and cDNA synthesis was done as previously described [19]. Briefly, RNA was isolated with the Nucleo Spin® RNA II Kit (Macherey-Nagel, Düren, Germany) and quantified with a NanoDrop1000 Spectrophotometer (Thermo Scientific, Bonn, Germany). The cDNA for real-time RT-PCR was prepared by using the SensiFAST cDNA Synthesis Kit (Bioline, Luckenwalde, Germany) from 200 ng RNA.

### Microarray analysis

For mRNA microarray analysis RNA Integrity Numbers (RIN) were determined in a Bioanalyzer System (Agilent Technologies Inc.). Numbers were 9.7 on average in both experimental groups (OA treated and vehicle treated GCs, 4 samples per group) ranging from 9.6 to 10.0. Total RNA of the different samples was hybridized on Bovine Gene 1.0 ST Array (Affymetrix, St. Clara, CA, USA). Amplification, labelling and hybridization was performed with the “GeneChip Expression 3’ Amplification One-Cycle Target Labeling and Control Reagents” (Affymetrix) according to the manufacturer’s protocol. Hybridization was done overnight in the GeneChipR Hybridization Oven (Affymetrix) and visualized with the Affymetrix GeneChip Scanner 3000. Raw data were processed with the Expression Console (V1.4.1.46; Affymetrix). Normalization, background reduction and a gene-level summary was done using the RMA method (Robust Multichip Average). Results of the array have been submitted to the GEO database (GSE152307). Subsequent analysis was done with the Transcriptome Analysis Console 3.0 (TAC3.0, Affymetrix) to check for differentially expressed genes under different conditions. Analysis of Variance (ANOVA) was used to calculate the *p*-value and was additionally corrected for FDR (False Discovery Rate, Benjamini-Hochberg method) integrated in TAC3.0. Levels of significance for differentially expressed genes were set at fold change (FC) > |1.5|, ANOVA *p* < 0.05 and FDR < 0.05.

### Real-time RT-PCR

Validation of microarray data was carried out by analysing the transcript abundance of four up-regulated (*ALDH1A1, CD36, SLC38A4, TGFB2 and PTSG2*), four down-regulated (*CYP19A1*, *NOS2*, *SERPINE2*, *FSHR*, *LHCGR*) and two unchanged genes (*CDH1* and *CDH2*) by real time RT-PCR. For this, 0.25 and 0.5 μl cDNA of each sample were amplified using SensiFastTM SYBR No-ROX (Bioline, Luckenwalde, Germany) and gene-specific primers (Supplementary Table 5) in a LightCycler 96 instrument (Roche). To ensure amplification of the correct products melting points of products were routinely analysed. Initially, all amplicons were cloned and sequenced. These cloned plasmids were used as external standards. For preparation of an external standard curve, five fresh dilutions were prepared with concentrations from 5 × 10^12^ to 5 × 10^16^ g DNA/reaction. These were co-amplified with each run of samples. After amplification, values of 0.25 and 0.5 μl of cDNA were averaged considering different dilutions. The size of products was routinely controlled by agarose gel electrophoresis. For normalization, the abundance of target transcripts was divided by the corresponding abundance of TBP housekeeping control transcripts [45].

### Bioinformatic analysis of differentially expressed genes

To identify “Upstream Regulators”, “Canonical Pathways”, and “Functions and Diseases” affected by OA treatment, differentially expressed genes (DEGs) were analyzed by the Ingenuity Pathway Analysis tool (IPA, QIAGEN, Hilden, Germany). In addition to this, gene enrichment and gene ontology analysis was done with the WEB-based GEneSeTAnaLysis Toolkit (WebGestalt).

### Quantification of 17-β-estradiol

The levels of E2 in conditioned media collected at different days after OA treatment were estimated with an ultrasensitive 125I-RIA (DSL, Sinsheim, Germany) in 200-μL duplicates. The standard curve was established between 0.005 and 0.750 ngmL− 1. Radioactivity was measured with an automatic gamma counter with integrated RIA calculation (Wizard; Perkin Elmer, Rodgau, Germany). The detection limit of the method was found at 0.003 ngmL− 1. The intra- and inter-assay CVs were 8.8 and 9.7%, respectively. The analysis of media was done with 10 μl of the undiluted sample in duplicates as also described in previous studies [17, 46].

### Statistical analysis

All experiments were carried out at least three times independently. Comparative data from OA effects on transcript abundance at different days were analysed by one-way analysis of variance (ANOVA) using the Holm-Sidak all pairwise multiple comparison procedure with the Sigma Plot 11.0 Statistical Analysis System (Jandel Scientific, San Rafael, CA, USA). Microarray re-evaluation data at the 8th day in culture were analysed by the paired t-test using the GraphPad prism 5.0 software. *P* values < 0.05 were considered significant.

## Supplementary Information

**Additional file 1: Figure S1.** GO terms associated with OA down-regulated genes according to WebGestalt (significantly affected GO term in red).

**Additional file 2: Figure S2.** GO terms associated with OA up-regulated genes according to WebGestalt (significantly affected GO term in red).

**Additional file 3: Table S1.** List of OA regulated genes (DEGs).

**Additional file 4: Table S2.** OA regulated Upstream Regulators according to IPA analysis.

**Additional file 5: Table S3.** OA regulated Canonical Pathways according to IPA analysis.

**Additional file 6: Table S4.** OA regulated Diseases and Functions according to IPA analysis.

**Additional file 7: Table S5.** List of primers used for transcript quantification by real-time RT-PCR.

## Data Availability

The datasets generated and/or analysed during the current study are available in the GEO repository, https://www.ncbi.nlm.nih.gov/geo/query/acc.cgi?acc=GSE152307 Primers for Real -time RT-PCR analysis were designed from published sequences with accession Nos: NM_174239.2, NM_001076372, NM_001278621.1, NM_001002763.1, NM_001166492.1, NM_174445, NM_174305, NM_174061, NM_174381, NM_174094.4, NM_001076799.1, NM_174669.2, NM_001205943.1, NM_001113252.1, NM_001075742.1.

## Abbreviations

DEG: Differentially Expressed Gene
E2: 17-β-Estradiol
EMT: Epithelial-to-Mesenchymal Transition
FFA: Free Fatty Acid
FSH: Follicle Stimulating Hormone
GC: Granulosa Cell
GO: Gene Ontology
IGF-1: Insulin-like-Growth Factor 1
IPA: Ingenuity Pathway Analysis
LH: Luteinizing Hormone
NEB: Negative Energy Balance
NEFA: Non-Esterified Fatty Acid
OA: Oleic Acid
P4: Progesterone
PA: Palmitic Acid
SA: Stearic Acid
