# Correction to: Genome wide effects of oleic acid on cultured bovine granulosa cells: evidence for the activation of pathways favoring folliculo-luteal transition

**DOI:** 10.1186/s12864-021-07933-3

**Published:** 2021-08-24

**Authors:** Vengala Rao Yenuganti, Dirk Koczan, Jens Vanselow

**Affiliations:** 1 Animal Biology Department, School of Life Sciences, University of Hyderabad, Hyderabad, Telagana India; 2 Institute for Immunology, University of Rostock, 18055 Rostock, Germany; 3 Institute of Reproductive Biology, Leibniz Institute for Farm Animal Biology (FBN), Wilhelm-Stahl-Allee 2, 18196 Dummerstorf, Germany

**Correction to: BMC Genomics 22, 486 (2021)**

**https://doi.org/10.1186/s12864-021-07817-6**

Following publication of the original article [1], it was reported that affiliations 1 and 3 were erroneously reversed. The affiliations have been correctly assigned for Vengala Rao Yenuganti and Jens Vanselow in this Correction article and the original article has been updated.
