# Supplementary figures and images for: Genome wide effects of oleic acid on cultured bovine granulosa cells: evidence for the activation of pathways favoring folliculo-luteal transition
Source: BMC Genomics. 2021 Jun 29;22:486. doi: 10.1186/s12864-021-07817-6 (PMC8243882; doi:10.1186/s12864-021-07817-6)

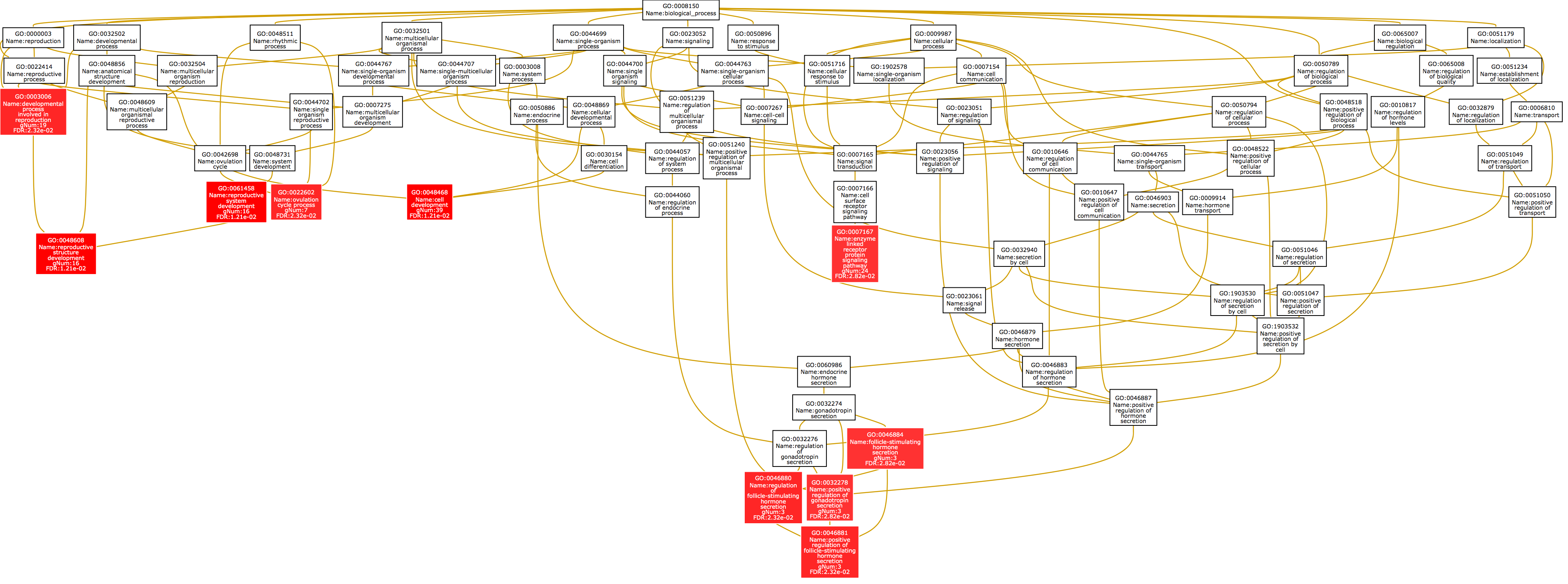

Supplement: Supplementary file 1 — Additional file 1: Figure S1. GO terms associated with OA down-regulated genes according to WebGestalt (significantly affected GO term in red). [file 12864_2021_7817_MOESM1_ESM.png]

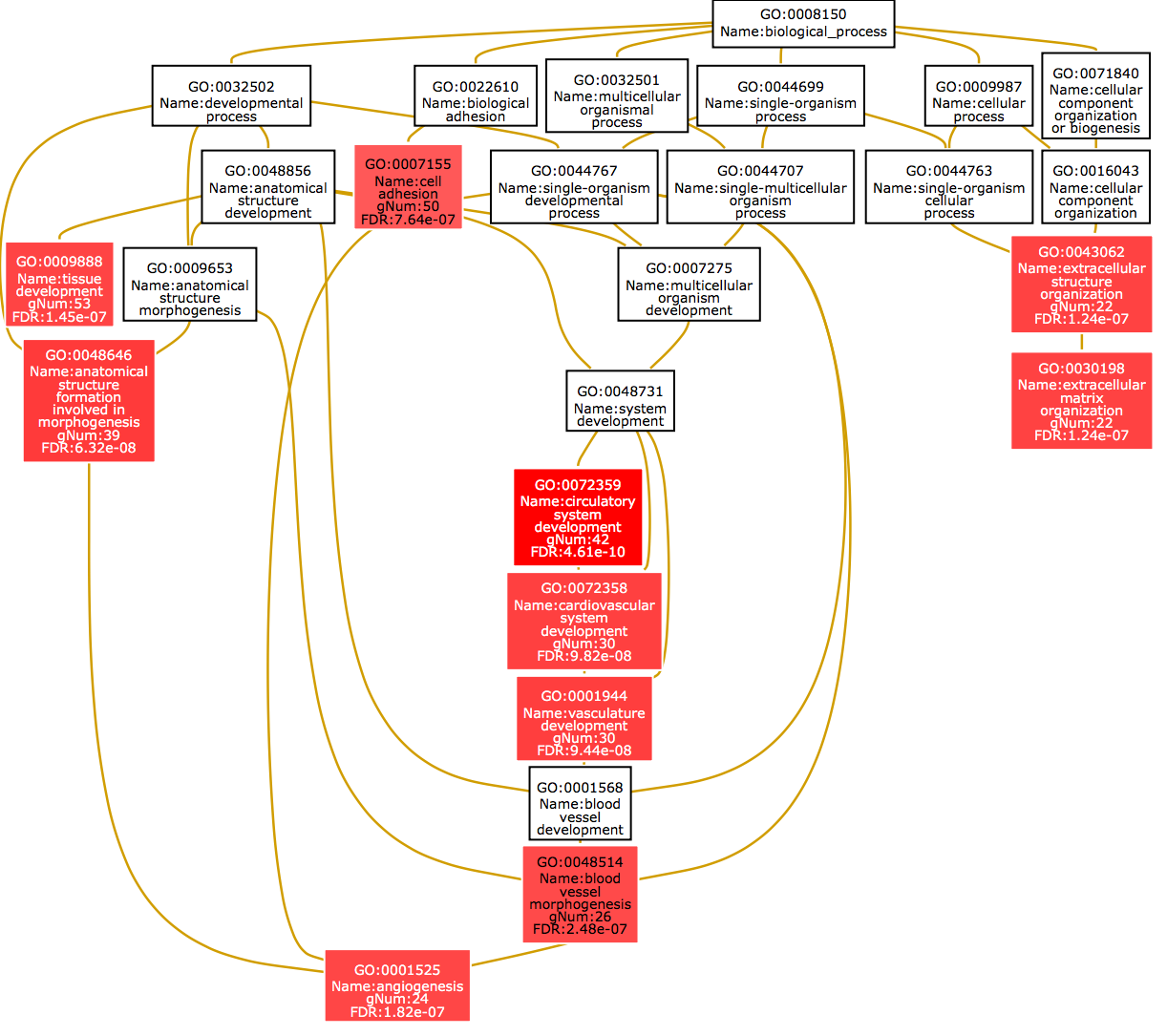

Supplement: Supplementary file 2 — Additional file 2: Figure S2. GO terms associated with OA up-regulated genes according to WebGestalt (significantly affected GO term in red). [file 12864_2021_7817_MOESM2_ESM.png]
