# Supplementary material for: Genome wide effects of oleic acid on cultured bovine granulosa cells: evidence for the activation of pathways favoring folliculo-luteal transition
Source: BMC Genomics. 2021 Jun 29;22:486. doi: 10.1186/s12864-021-07817-6 (PMC8243882; doi:10.1186/s12864-021-07817-6)
Supplement: Supplementary file 7 — Additional file 7: Table S5. List of primers used for transcript quantification by real-time RT-PCR. [file 12864_2021_7817_MOESM7_ESM.docx]

**Supplementary Table 5. List of primers used for transcript quantification by qPCR.**

| **Name** | **Sequence** | **Size (bp)** | **Accession no.** |
| --- | --- | --- | --- |
| *AlDH1A1*Forward | CAAACCAGCAGAGCAAACCC | 180 | NM_174239.2 |
| *AlDH1A1*Reverse | TTGCCAACCTCTGTCGATCC |  |  |
| *CCND2* Forward | CGCAGGGCCGTGCCGGACGCCAAC | 279 | NM_001076372**^a^** |
| *CCND2* Reverse | CACGGCCCCCAGCAGCTGCAGATGG |  |  |
| *CD36* Forward | GCTCCTTAAGCCATTCTTGGAT | 151 | NM_001278621.1**^a^** |
| *CD36* Reverse | CACCAGTGTCAACGCACTTT |  |  |
| *CDH1* Forward | GGCTGGACCGTGAGAGTTTT | 188 | NM_001002763.1 |
| *CDH1* Reverse | GGTGATGGCCACATTAGCCT |  |  |
| *CDH2* Forward | CCGATCCTGCAAACTGGCTA | 136 | NM_001166492.1 |
| *CDH2* Reverse | GGGATTCCATTGTCAGAGGCA |  |  |
| *PTGS2* Forward | TACAGCACTTGAGTGGCTATCAC | 317 | NM_174445 |
| *PTGS2* Reverse | CTGGTCAATTGAAGCCTTTGATAC |  |  |
| *CYP19A1 F*orward | GCTTTTGGAAGTGCTGAACCCAAGG | 172 | NM_174305**^a^** |
| *CYP19A1* Reverse | GGGCCCAATTCCCAGAAAGTAGCTG |  |  |
| *FSHR* Forward | TCACCAAGCTTCGAGTCATCCCAAA | 189 | NM_174061**^a^** |
| *FSHR* Reverse | TCTGGAAGGCATCAGGGTCGATGTA |  |  |
| *LHCGR* Forward | GCATCCACAAGCTTCCAGATGTTACGA | 205 | NM_174381**^a^** |
| *LHCGR* Reverse | GGGAAATCAGCGTTGTCCCATTGA |  |  |
| *INHA* Forward | AGGAGGGCCTCTTCACGTATG | 94 | NM_174094.4**^b^** |
| *INHA* Reverse | TCCAGTCCTGTGTGGAACCA |  |  |
| *NOS2* Forward | CATTCGATGTCAGCGGCAAG | 131 | NM_001076799.1 |
| *NOS2* Reverse | ACTGCTCAGGGCTTTGTTGA |  |  |
| *SERPINE2* Forward | CGAGACGCAGGGTATGATCG | 150 | NM_174669.2 |
| *SERPINE2* Reverse | GCCACAAATGTGCGCTTCTT |  |  |
| *SLC38A4* Forward | GACACCCCTCTCCTCATGGT | 234 | NM_001205943.1 |
| *SLC38A4* Reverse | GGCAGAAGAAGCCCCTATGAAT |  |  |
| *TGFB2* Forward | ACCGGCGGAAGAAGCGCGCTCTGGA | 257 | NM_001113252.1 |
| *TGFB2* Reverse | GGACACGCAGCAAGGGGAAGCAGACGC |  |  |
| *TBP* Forward | GCCTTGTGCTTACCCACCAACAGTTC | 200 | NM_001075742.1**^a^** |
| *TBP* Reverse | TGTCTTCCTGAAACCCTTCAGAATAGGG |  |  |

**a=** primers from Yenuganti et al., 2016, **b**=primer from Sugawara et al., 2010.
